# Supplementary material for: Abdominal Photobiomodulation and the Gut-Brain Axis: A Systematic Review of Mechanistic and Translational Evidence
Source: Biomedicines. 2025 Dec 11;13(12):3042. doi: 10.3390/biomedicines13123042 (PMC12730906; doi:10.3390/biomedicines13123042)
Supplement: Supplementary file 1 [file biomedicines-13-03042-s001.zip › Table S1_Guimaraes(2025).pdf]

**Table S1. Summary of Clinical Studies investigating abdominal PBM in humans**					
Study	***Design***	***Sample***	*Condition*	***PBM Protocol***	***Main Outcomes***
Liebert et al. (2022)	Prospective, single-arm cases series	Adults = 7 M<br>Mean age = 64.4 yrs	Parkinson's Disease Stages I-III<br>Stable medication	• 904 nm, class 1- 4/2 diodes.<br>• Abdomen (9 points), C1/C2.<br>• 12 weeks in clinic (3×/week, 10 min/day), followed by 33 weeks home-based (3×/week, 20 min/day).	• Improvements in dynamic balance, mobility (TUG, gait speed), fine motor skills, and cognition after 12 weeks, with TUG and cognitive gains sustained in several participants at 45 weeks. Improvement in anosmia in two participants.<br>• No deterioration over one year.<br>• No microbiota analysis.
Bicknell & Laakso et al. (2022)	Single-subject case report	Adult = 1 female<br>Age = 57 yrs	Breast cancer & Obesity	• 904 nm, 700 Hz, 12-diode abdominal laser.<br>• 20 min, 3×/week, 11 weeks	• Improved alpha diversity.<br>• ↑ *Akkermansia*, ↑ *Faecalibacterium*, ↑ *Roseburia*.<br>• ↓ Proteobacteria.<br>• Improved Firmicutes: Bacteroidetes ratio.
Bicknell et al. (2022)	Non-randomized, retrospective clinical study	Adults = 12 (5M/7F)<br>Mean age = 70.8 yrs	Parkinson's Disease Stages I–III	• 904 nm device<br>• 24 clinic sessions (3→1×/week), 30 min/day.<br>• Same device as used in Bicknell & Laakso et al. (2022).	• No significant changes in α or β diversity.<br>• ↓ Firmicutes, ↑ Bacteroidetes, Verrucomicrobia.<br>• 9/12 showed improved Firmicutes: Bacteroidetes ratio.<br>• No clinical scales used.
Blivet et al. (2022)	Randomized, double-blind, sham-controlled pilot trial	Adults = 53 (31F/22M)<br>Mean age = 73 yrs	Alzheimer's Disease MMSE ≥ 21	• Helmet + abdominal belt: 3× LEDs (660/850 nm) + 3× 850 nm lasers.<br>• 10 Hz pulsed magnetic field.<br>• 25 min/day, 5×/week, 8 weeks.	• Selective cognitive gains in language, memory, executive function (TMT-B).<br>• No significant MMSE changes.<br>• No serious adverse events.<br>• No microbiota data.
Liebert et al. (2024)	Long-term open-label extension study – 5 years	Adults = 7<br>Continued from Bicknell et al. (2022) cohort.	Parkinson's Disease Stages I–III	• 904 nm device<br>• 12 weeks in clinic, followed by 33 weeks home-based (3×/week, 30 min), maintained up to 5 years.<br>• Same device as used in Bicknell et al. (2022).	• Improved or stable cognition, motor function, balance, olfaction, and quality of life.<br>• No adverse events.<br>• No microbiota data.

Table S1. Summary of clinical studies investigating abdominal PBM in humans.

Legend. Overview of clinical studies evaluating abdominal PBM in patients with Parkinson's disease, Alzheimer's disease, or cancer-related dysbiosis. Devices combined abdominal laser delivery (904–850 nm) with optional transcranial, cervical, or intranasal stimulation. PD: Parkinson's disease; AD: Alzheimer's disease; MMSE: Mini-Mental State Examination; TMT-B: Trail Making Test B; AEs: adverse events, TUG: Time Up and Go test.
