# Supplementary material for: Abdominal Photobiomodulation and the Gut-Brain Axis: A Systematic Review of Mechanistic and Translational Evidence
Source: Biomedicines. 2025 Dec 11;13(12):3042. doi: 10.3390/biomedicines13123042 (PMC12730906; doi:10.3390/biomedicines13123042)
Supplement: Supplementary file 1 [file biomedicines-13-03042-s001.zip › Table S2_Guimaraes(2025).pdf]

Table S2. Summary of Animal Studies					
Study	***Design***	***Animal Model & Sample Size***	***PBM Protocol***	***Assessments***	***Main Findings***
Bicknell et al. (2019)	Experimental, sham-controlled	Healthy mice<br>n = 4/group	• 660 or 808 nm laser<br>• fluence 10 J/cm²<br>• 0.8 cm² spot size<br>• 250 Hz<br>• 1× or 3×/week for 2 weeks.<br>• Applied to shaved abdomen.	• 16S rRNA sequencing of fecal samples on days 0, 7, and 14.	• PBM altered gut microbiota composition.<br>• 808 nm PBM increased *Allobaculum* abundance ($p<0.001$), associated with healthy gut profile.
Chen et al. (2021)	Randomized, sham-controlled	C57BL/6N mice<br>n = 15/group	• 630, 730, or 850 nm LED<br>• 100 J/cm²<br>• 10 mW/cm²<br>• 1000 s/day<br>• 5×/week for 8 weeks.<br>• Irradiation to upper abdomen.	• Morris water maze<br>• Hippocampal immunofluorescence<br>• Proteomics<br>• Gut microbiota (16S)	• PBM improved learning/memory, reduced Aβ and tau, and normalized hippocampal protein profiles.<br>• Reversed dysbiosis (*Helicobacter*, *Bacteroidales*↓; *Rikenella*↑).
Gordon et al. (2023)	Randomized, blinded	Macaques, n=3<br>Mice, n=8-10/group	• 670 nm (macaques), 656 nm (mice)<br>• 50 mW/cm²<br>• 9 J/cm² (abdomen/head), 4.5 J/cm² (legs)<br>• Daily for 5 days (primates) or 21 days (mice).	• Clinical scoring<br>• Pole test<br>• TH+ and Nissl staining<br>• Striatal terminal density (ImageJ/Fiji)	• Abdominal PBM preserved dopaminergic neurons (~50% TH+ in SNc), improved motor scores, and outperformed transcranial PBM.
Sancho-Balsells et al. (2024)	Chronic stress model (CUMS)	C57BL/6 mice<br>n=3–5/group	• Mixed LED/laser (630 + 850 nm) pulsed at 10 Hz<br>• With magnetic field (200 mT).<br>• 6 min/day × 3 weeks.<br>• Applied to abdomen, head, or both.	• Behavioral tests (Y-maze, NOL, forced swim)<br>• Hippocampal histology<br>• Glial markers<br>• 16S rRNA (gut)	• Combined PBM (gut+brain) improved cognition, reduced IL-6/TNF-α, increased Sirt1 and dendritic spine density, and reversed dysbiosis (*Roseburia*↑).<br>• Synergistic effects noted.

Table S2. Summary of preclinical studies investigating abdominal PBM and gut-brain axis interactions.

Legend. Overview of animal studies evaluating the effects of abdominal PBM on gut-brain axis outcomes. All studies applied red or near-infrared light to the abdominal region and assessed effects on microbiota, neuroinflammation, behavior, or neuronal integrity. CUMS: chronic unpredictable mild stress; SNc: substantia nigra pars compacta; TH^+: tyrosine hydroxylase-positive neurons; NOL: novel object location.
