# Supplementary material for: Abdominal Photobiomodulation and the Gut-Brain Axis: A Systematic Review of Mechanistic and Translational Evidence
Source: Biomedicines. 2025 Dec 11;13(12):3042. doi: 10.3390/biomedicines13123042 (PMC12730906; doi:10.3390/biomedicines13123042)
Supplement: Supplementary file 1 [file biomedicines-13-03042-s001.zip › Table_S3_Guimaraes(2025).pdf]

Table S3. Search strategy.

Database	Search String	Limits
MEDLINE (PubMed; last searched 15-May-2025)	("photobiomodulation" OR "low-level light therapy" OR PBM OR LLLT) AND (abdomen OR abdominal OR "abdominal application" OR "gut-brain axis" OR "enteric nervous system") AND (microbiome OR microbiota OR "gut bacteria" OR "intestinal flora" OR microbial)	Publication type: Case report, Clinical Study, Observational Study, Randomized Controlled Trial. Population: Humans, Animals. Language: English, Spanish, Portuguese. Date: None (from inception to 15-May-2025).
Scopus (Elsevier; last searched 15-May-2025)	TITLE-ABS-KEY (photobiomodulation OR "low-level light therapy" OR PBM OR LLLT) AND TITLE-ABS-KEY (abdomen OR abdominal OR "gut-brain axis" OR "enteric nervous system") AND TITLE-ABS-KEY (microbiome OR microbiota OR "intestinal flora" OR "gut bacteria")	Publication type: Case report, Clinical Study, Observational Study, Randomized Controlled Trial. Population: Humans, Animals. Language: English, Spanish, Portuguese. Date: None (to 15-May-2025).
ScienceDirect (Elsevier; last searched 15-May-2025)	All fields: (photobiomodulation OR "low-level light therapy" OR PBM OR LLLT) AND (abdomen OR abdominal OR "gut-brain axis" OR "enteric nervous system") AND (microbiome OR microbiota OR "intestinal flora" OR "gut bacteria")	Publication type: Case report, Clinical Study, Observational Study, Randomized Controlled Trial. Population: Humans, Animals. Language: English, Spanish, Portuguese. Date: None (to 15-May-2025).
Web of Science Core Collection (Clarivate; last searched 16-May-2025)	TS=(photobiomodulation OR "low-level light therapy" OR PBM OR LLLT) AND TS=(abdomen OR abdominal OR "gut-brain axis" OR "enteric nervous system") AND TS=(microbiome OR microbiota OR "intestinal flora" OR "gut bacteria")	Indexes: SCI-EXPANDED, SSCI, ESCI (as available). Publication type: Case report, Clinical Study, Observational Study, Randomized Controlled Trial. Population: Humans, Animals. Language: English, Spanish, Portuguese. Date: None (to 16-May-2025).

Note: All databases were searched using the same limits described above, and the reference lists of included studies were also screened. All search strings were pilot-tested and refined to balance sensitivity and specificity, and the exact Boolean logic and field tags used in MEDLINE are provided in Appendix A to ensure full reproducibility.
